# Supplementary material for: Sc-compReg enables the comparison of gene regulatory networks between conditions using single-cell data
Source: Nat Commun. 2021 Aug 6;12:4763. doi: 10.1038/s41467-021-25089-2 (PMC8346476; doi:10.1038/s41467-021-25089-2)
Supplement: Supplementary file 1 — Supplementary Information [file 41467_2021_25089_MOESM1_ESM.pdf]

Supplementary note: Sc-compReg enables the comparison of gene regulatory networks between conditions using single-cell data

Duren et. al

Supplementary note S1: Bad cell identification

We cluster the scATAC-seq data into 7 clusters by non-negative matrix factorization (NMF). The results show one of the clusters (cluster 7) has 8.63-fold higher sequencing depth than others (Supplementary Figure 1C-E) and has a much lower percentage of reads in peak (Supplementary Figure 1F). The cells in cluster 7 are closed to each other in t-SNE (Supplementary Figure S1D). The distribution of reads over chromosomes is also different from other clusters (Supplementary Figure S2). These observations suggest those cells may be dead or dying cells. Based on the ground truth, cluster 7 contains all 13 cell types present in the sample (Fig. 3A). These results suggest that the clustering of scATAC-seq may be affected by the sequencing depth and percentage of reads in peaks. Hence, we exclude these outlier cells from all subsequent analyses.

Supplementary note S2: Comparison of peak calling methods in terms of clustering

We cluster the cells based on the accessibility of peaks from MACS2 and peaks from the 10X peak caller. The results show that clustering based on 10X peak caller are much more consistent with the ground truth. Our results also suggest that running NMF multiple times and doing consistency clustering improves the clustering performance (Supplementary Figure S3).

Supplementary note S3: Comparison of different clustering methods

CoupledNMF: We set K=7 and get well-separated clusters in t-SNE for both scRNA-seq and scATAC-seq from healthy donor BMMC data (Fig. 3A-B, right). In both scRNA-seq and scATAC-seq embeddings, clusters 1, 2, and 5 are close to each other; these correspond to CD4 T cells (CD4), CD8 T cells (CD8) and NK cells (NK) based on the ground truth label inferred from FACS-sorted data (Fig. 3A-B, left). Clusters 6 and 7 are different from all other clusters and correspond to Monocyte cells (Mono) and B cells (B), respectively. Cluster 3 is a minor population that corresponds to erythrocyte cells (Ery). The remaining cells are clustered into cluster 4, which corresponds to progenitor cells including MEP and CLP. On B cell and Mono, we get over 90% accuracy both on scRNA-seq and scATAC-seq. On average, the accuracy is 76.63% and 72.97% and for scRNA-seq and scATAC-seq respectively.

CellRanger and Seurat: We compare our results with those from two widely used single cell analysis softwares: CellRanger (10X genomics) and seurat. In CellRanger, we apply graph cluster as well as K-means (scRNA-seq) or K-medoids (scATAC-seq) with K ranging from 2 to 10 (Supplementary Figure S4-S5). Supplementary Figure S6 shows the results of the comparison. CoupledNMF outperforms all K-means (K=2~10), K-medoids (K=2~10) and graph clustering. In CellRanger, K-means/K-medoids with K=10 have the best performance. Fig. 3C shows the comparison results of three methods, CoupledNMF (K=7), Seurat version 3.1.1 (1), and CellRanger (K=10). First, we check their performance of the clustering on CD4, CD8, and NK, which are very similar and difficult to be distinguished. In CellRanger scRNA-seq clustering, we didn't find any clusters that are matched to CD8 cells. Among 203 CD8 cells, 65.57% and 29.72% are clustered into NK and CD4 clusters respectively. In Seurat scRNA-seq clustering, the accuracy of the NK cluster is 42.05%. The reason is

about 55.01% of the NK cells are clustered into CD8 clusters (Fig. 2C, Supplementary Figure S7). Our method CoupledNMF has better performance in all these three cell types with an accuracy of 75.64%, 68.40%, and 72.36% for CD4, CD8, and NK respectively. Next, we compare the clustering performance on scATAC-seq data. Seurat has almost 2-fold lower accuracy than CoupledNMF on Ery, NK, and Progenitor cells. CellRanger scATAC-seq clustering on CD4 and Progenitor cells have much lower accuracy (47.34% and 42.41%) than CoupledNMF (74.15% and 76.21%). Overall, the clusters from coupled clustering is highly consistent with the true labels and outperform other clustering methods CellRanger and Seurat.

Supplementary note S4: TOX2 motif

To do the regulatory analysis for TOX2, we detect the TOX family motif from the TOX dependent open regions in T cell (2) by motif enrichment. We assume TOX has a similar motif with TOX2 as 1) they have only 5 amino acids difference in 68 amino acids length DNA binding domain and 2) their target genes are significantly overlapped (Supplementary figure S11A-B). We find the most enriched motif is very similar to the known motif of TCFL2, which has the same DNA binding domain High Mobility Group (HMG) Box with the TOX family (Supplementary Figure S11C). This suggests that we have got a good TOX family motif by comparing wild type versus TOX knockout ATAC-seq data.

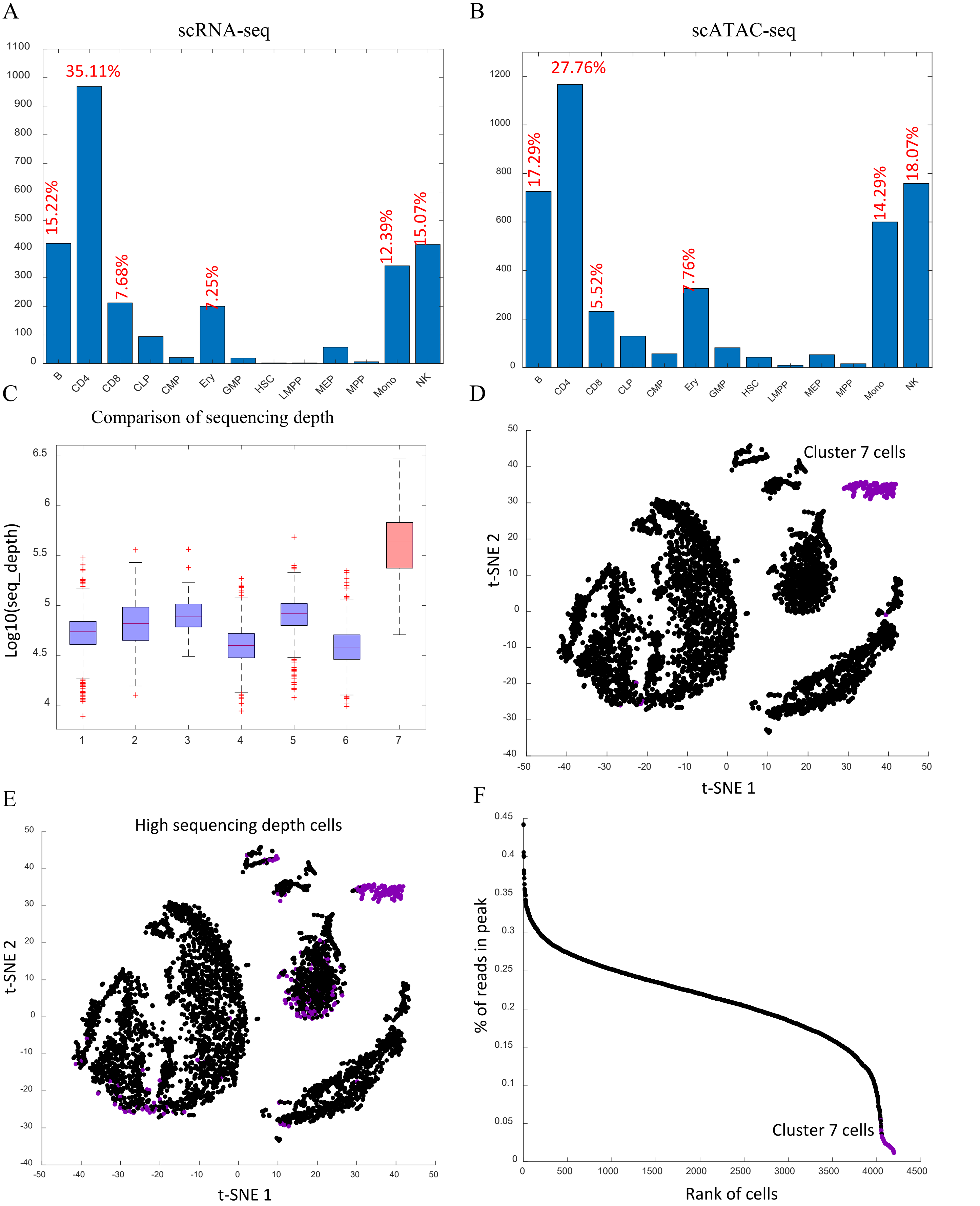

Fig. S1. Identification of bad cells in scATAC-seq. (A-B) The fraction of cell types in scRNA-seq data and scATAC-seq data based on ground truth. (C) Distribution of sequencing depth in different clusters by NMF (K=7). The number of cells in seven clusters are 1028, 311, 91, 1321, 530, 769, and 150 respectively. The central mark indicates the median, the bottom and top edges of the box indicate lower and upper quartiles, the whiskers extend to the most extreme data points not considered outliers, and the outliers are plotted individually using the '+' symbol. (D) t-SNE plot of scATAC-seq data colored by clustering label. The sequencing-depth dependent cluster (cluster 7) is labeled in purple color. (E) the t-SNE plot of scATAC-seq colored by outliers of distribution of log10 scale read counts. (F) Percentage of reads in peaks. The purple dots represent cells from cluster 7.

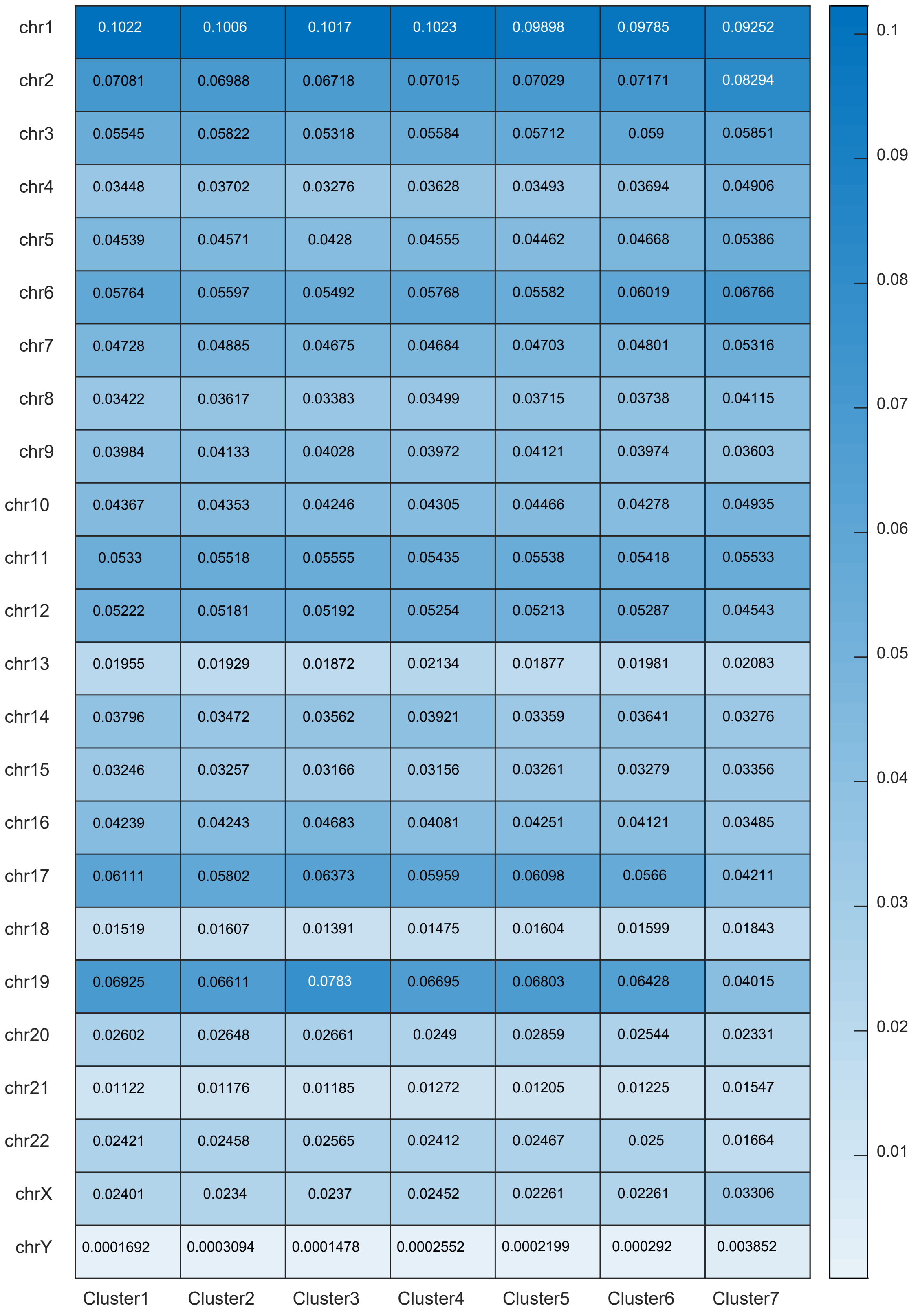

Fig. S2. The distribution of scATAC-seq reads over chromosomes on each cluster. Chr17, Chr19, and ChrX have a very different proportion of reads.

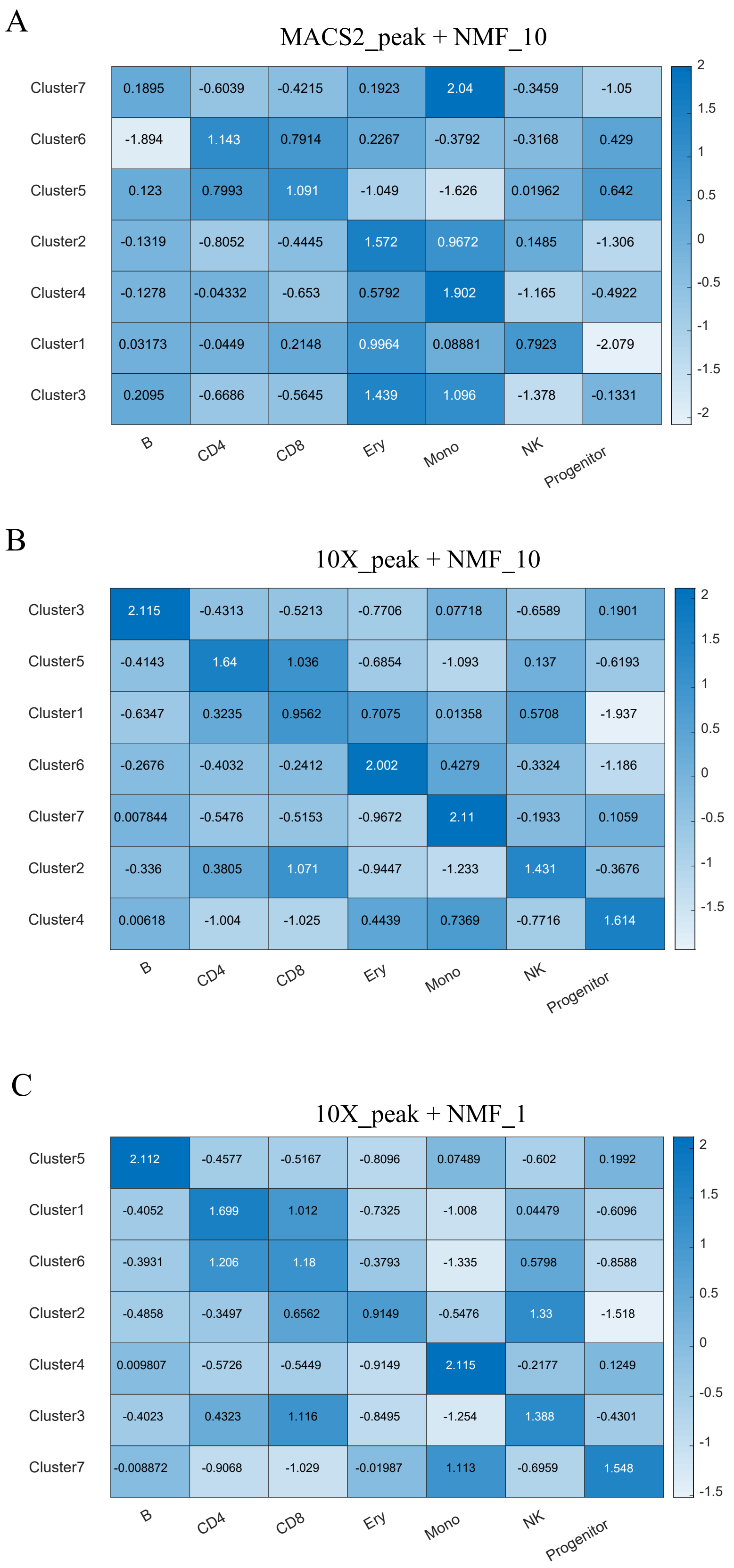

Fig. S3. Comparison of different peak calling methods.. (A-C) Mean accessibility of top 5000 cluster-specific peaks on FACS sorted bulk ATAC-seq data. The values represent z-score of mean accessibility across cell types. Clusters are one-to-one linked to the cell types by bipartite graph maximum weighted matching. (A) is based on MACS2 peaks, (B-C) are based on Cell Ranger peaks. (A-B) are the results of the consistency clustering of 10 runs of NMF. (C) is the result of one single NMF.

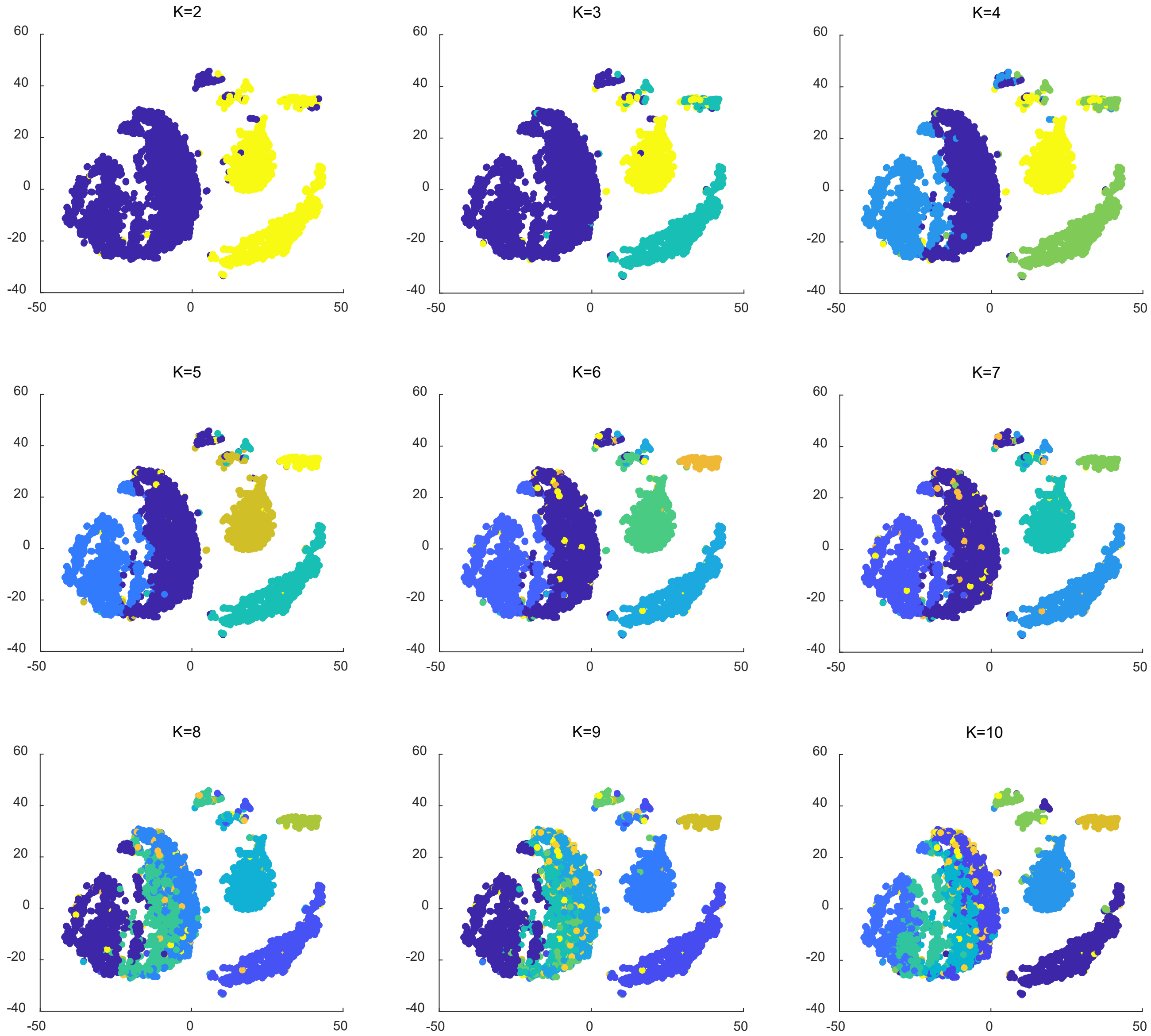

Fig. S4. Clustering results of Cell Ranger K-medoids method on scATAC-seq data. Each subplot represents the t-SNE plot with different K, where K is ranging from 2 to 10.

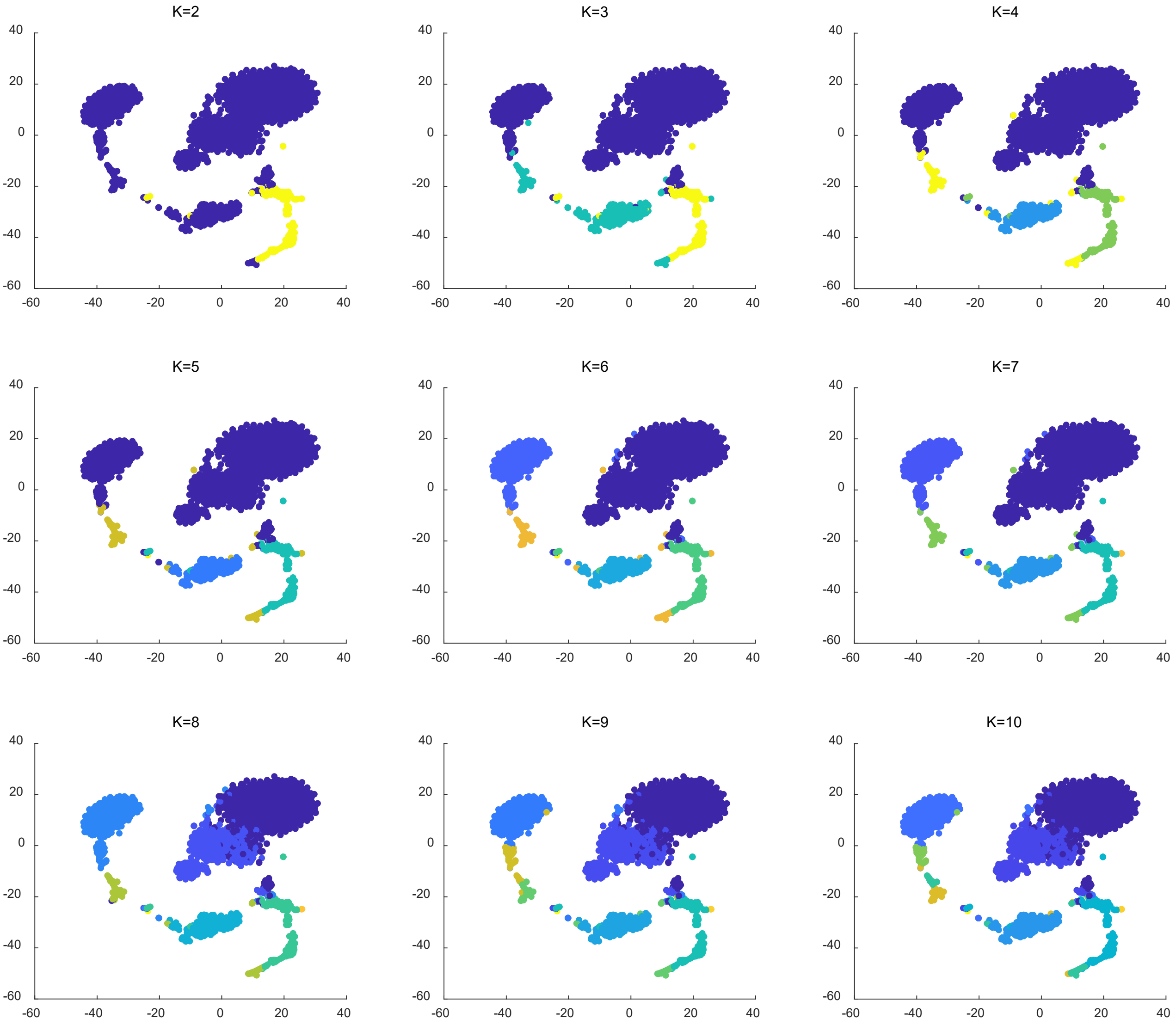

Fig. S5. Clustering results of Cell Ranger K-means method on scRNA-seq data. Each subplot represents the t-SNE plot with different K, where K is ranging from 2 to 10.

A

CellRanger_K7_RNA	B	CD4	CD8	Ery	Mono	NK	Progenitors
Cluster 1	415	9	0	0	7	2	61
Cluster 2	0	929	202	0	1	398	3
Cluster 3	0	0	0	0	1	0	0
Cluster 4	5	28	9	170	30	11	20
Cluster 5	0	1	1	0	300	1	3
Cluster 6	0	0	0	2	0	0	0
Cluster 7	0	2	0	28	3	4	114
Accuracy	0.9881	0.9587	0.0000	0.8500	0.8772	0.0000	0.5672

B

CellRanger_K7_ATAC	B	CD4	CD8	Ery	Mono	NK	Progenitors
Cluster 1	673	0	0	4	0	0	132
Cluster 2	5	1027	27	236	3	1	40
Cluster 3	2	29	4	3	5	8	6
Cluster 4	5	42	1	19	1	11	6
Cluster 5	0	0	0	5	509	0	96
Cluster 6	8	44	181	45	1	729	5
Cluster 7	0	3	0	3	3	1	5
Accuracy	0.9711	0.8969	0.0188	0.0603	0.9751	0.9720	0.0172

C

	ATAC_nmi	RNA_nmi	ATAC_mean_accuracy	RNA_mean_accuracy
K=2	0.5372	0.2099	0.2831	0.2687
K=3	0.6130	0.4414	0.4235	0.3890
K=4	0.7046	0.4829	0.5601	0.4710
K=5	0.7068	0.4822	0.5616	0.4706
K=6	0.6851	0.6478	0.5630	0.6066
K=7	0.6649	0.6473	0.5588	0.6059
K=8	0.6331	0.7131	0.5920	0.7294
K=9	0.6307	**0.7295**	0.5792	0.7115
K=10	0.6370	0.7236	0.6652	0.6907
graph cluster	0.6514	0.6425	0.6289	0.6550
CoupledNMF_7	**0.7126**	0.6898	**0.7297**	**0.7663**

Fig. S6. Performance of different methods with different parameters from Cell Ranger software on scATAC-seq and scRNA-seq data. (A-B) Linking of clusters to the true label by bipartite graph maximum weighted matching. (C) Comparison of mean accuracy (over cell types) and normalized mutual information of different clustering methods, including K-means/K-medoids with different K, graph clustering, and CoupledNMF.

A, CellRanger (K=10)

scRNA-seq

	B	CD4	CD8	Ery	Mono	NK	Progenitors
Cluster 1	404	9	0	0	7	2	8
Cluster 2	0	892	63	0	2	6	2
Cluster 3	0	2	0	34	2	1	48
Cluster 4	5	28	9	164	30	11	20
Cluster 5	0	1	1	0	299	1	3
Cluster 6	0	37	139	0	1	395	2
Cluster 7	0	0	0	0	0	0	62
Cluster 8	11	0	0	0	0	0	56
Cluster 9	0	0	0	2	0	0	0
Cluster 10	0	0	0	0	1	0	0
Accuracy	0.9619	0.9205	0.0000	0.8200	0.8743	0.9495	0.3085

scATAC-seq

	B	CD4	CD8	Ery	Mono	NK	Progenitors
Cluster 1	676	0	0	2	0	0	113
Cluster 2	2	542	11	69	0	0	11
Cluster 3	6	46	152	35	0	202	4
Cluster 4	2	401	10	118	4	1	7
Cluster 5	0	0	0	4	512	0	21
Cluster 6	2	3	35	12	2	536	1
Cluster 7	0	0	0	53	0	0	123
Cluster 8	0	2	0	1	3	0	4
Cluster 9	0	115	4	12	0	2	2
Cluster 10	5	36	1	9	1	9	4
Accuracy	0.9755	0.4734	0.7136	0.3746	0.9808	0.7147	0.4241

B, Seurat

scRNA-seq

	B	CD4	CD8	Ery	Mono	NK	Progenitor
Cluster 3	394	11	0	0	8	2	6
Cluster 1	0	807	17	0	0	2	2
Cluster 2	0	110	182	0	0	225	0
Cluster 6	0	0	0	147	0	0	18
Cluster 4	0	1	1	0	265	1	2
Cluster 5	0	0	1	0	0	172	0
Cluster 7	0	2	0	2	3	4	89
Cluster 8	19	1	0	0	0	0	58
Cluster 9	6	19	3	2	12	3	9
Cluster 10	0	0	0	0	36	0	3
Accuracy	0.9403	0.8486	0.8922	0.9735	0.8179	0.4205	0.4759

scATAC-seq

	B	CD4	CD8	Ery	Mono	NK	Progenitor
Cluster 3	667	3	1	2	0	0	40
Cluster 1	3	1071	32	182	1	5	17
Cluster 2	2	56	167	46	0	457	3
Cluster 6	0	0	0	58	1	0	13
Cluster 4	0	0	0	6	517	0	21
Cluster 5	2	4	13	5	1	288	1
Cluster 7	9	0	0	0	1	0	105
Cluster 8	1	11	0	16	0	0	88
Cluster 9	9	0	0	0	0	0	2
Cluster 10	0	0	0	0	1	0	0
Accuracy	0.9625	0.9354	0.7840	0.1841	0.9904	0.3840	0.3621

C, CoupledNMF

scRNA-seq

	B	CD4	CD8	Ery	Mono	NK	Progenitor
Cluster 7	411	7	0	0	1	0	48
Cluster 1	0	733	9	0	0	0	0
Cluster 2	1	176	145	5	5	112	14
Cluster 3	0	1	1	169	3	1	6
Cluster 6	0	2	1	0	308	2	6
Cluster 5	0	37	55	0	1	301	0
Cluster 4	8	13	1	26	24	0	127
Accuracy	0.9786	0.7564	0.6840	0.8450	0.9006	0.7236	0.6318

scATAC-seq

	B	CD4	CD8	Ery	Mono	NK	Progenitor
Cluster 7	664	0	0	0	2	1	39
Cluster 1	2	849	22	129	4	0	16
Cluster 2	5	239	138	33	1	166	3
Cluster 3	3	51	6	124	8	17	6
Cluster 6	0	0	0	0	479	0	4
Cluster 5	3	4	47	14	2	561	1
Cluster 4	16	2	0	15	26	5	221
Accuracy	0.9582	0.7415	0.6479	0.3937	0.9176	0.7480	0.7621

Fig. S7. Linking of clusters to the true label by bipartite graph maximum weighted matching. Here we compare three different methods (A) Cell Ranger, (B) Seurat, and (C) CoupledNMF.

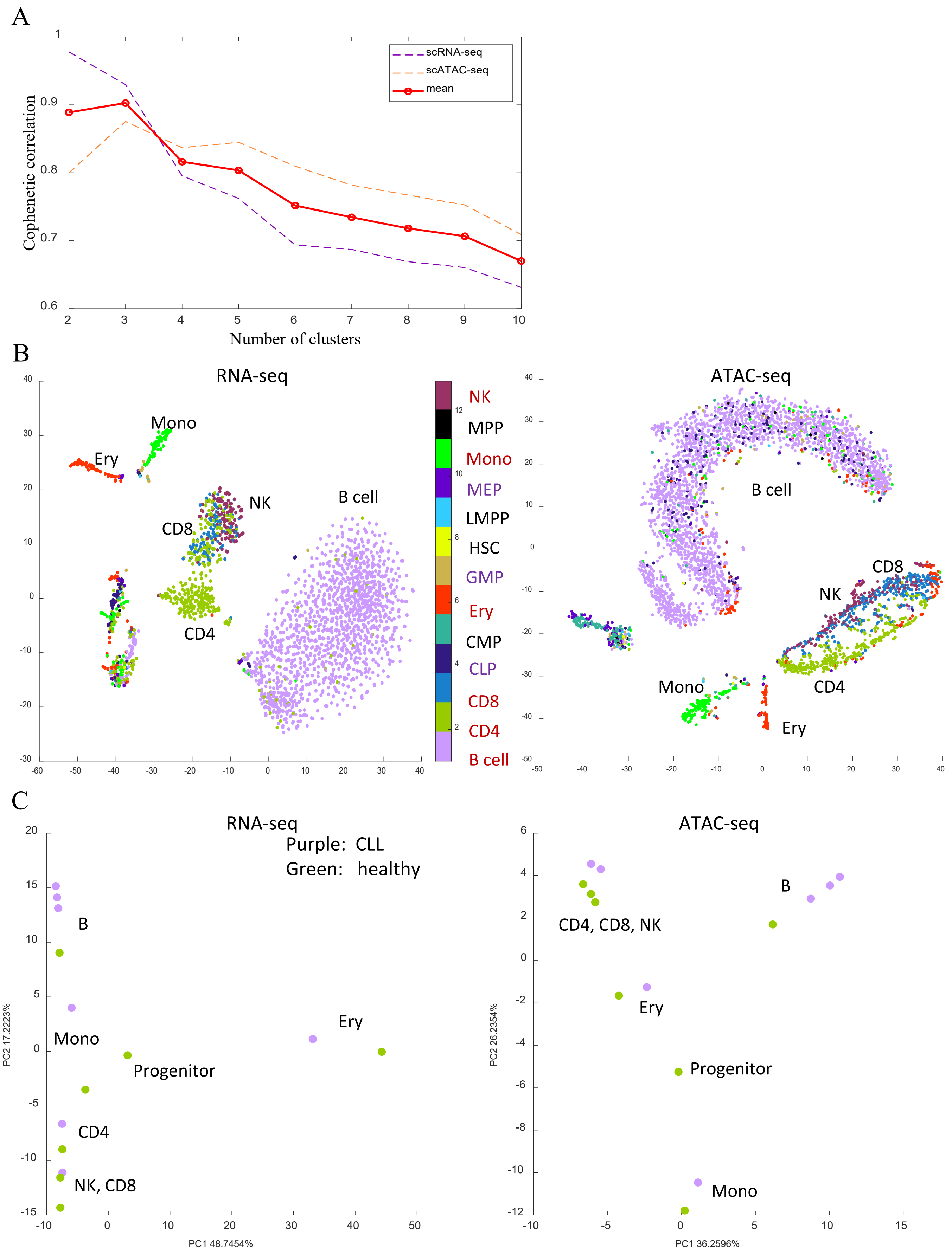

Fig. S8. Analysis of single cell transcriptomic and epigenomic data from CLL donor. (A) Clustering stability of B cells' scRNA-seq and scATAC-seq from CLL donor. (B) the t-SNE plot of scATAC-seq (right) and scRNA-seq (left) from CLL donor colored with FACS sorting based ground truth. (C) Comparison of scRNA-seq and scATAC-seq clustering profile between the healthy donor and CLL donor by principal component analysis (PCA).

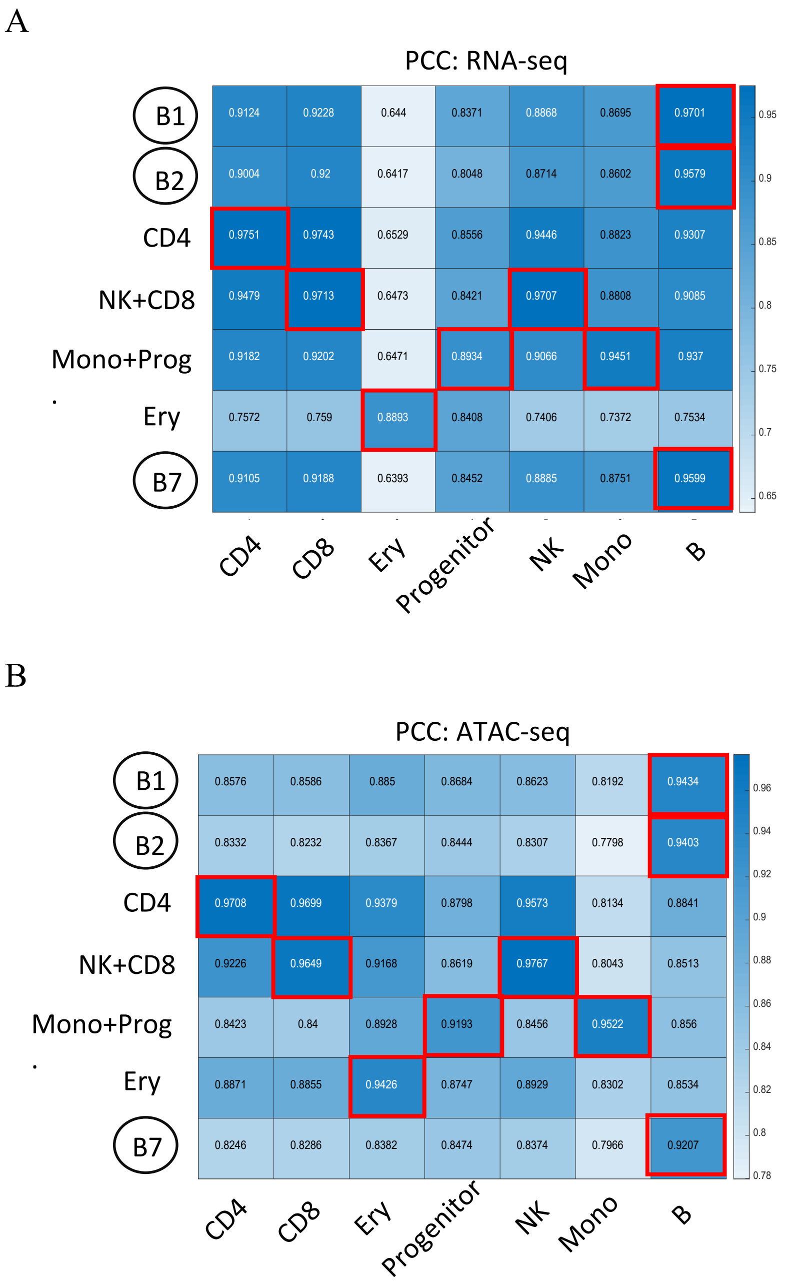

Fig. S9. Pearson correlation coefficient (PCC) of seven cluster’s expression and accessibility profile with FACS sorted bulk data. (A) PCC on scRNA-seq. (B) PCC on scATAC-seq.

A

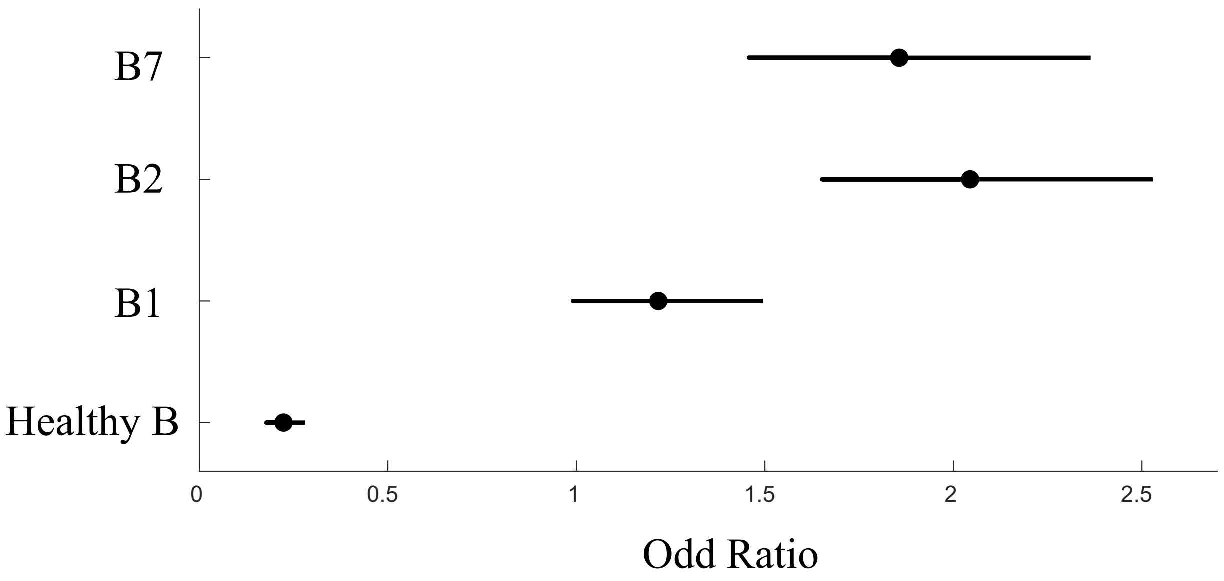

B

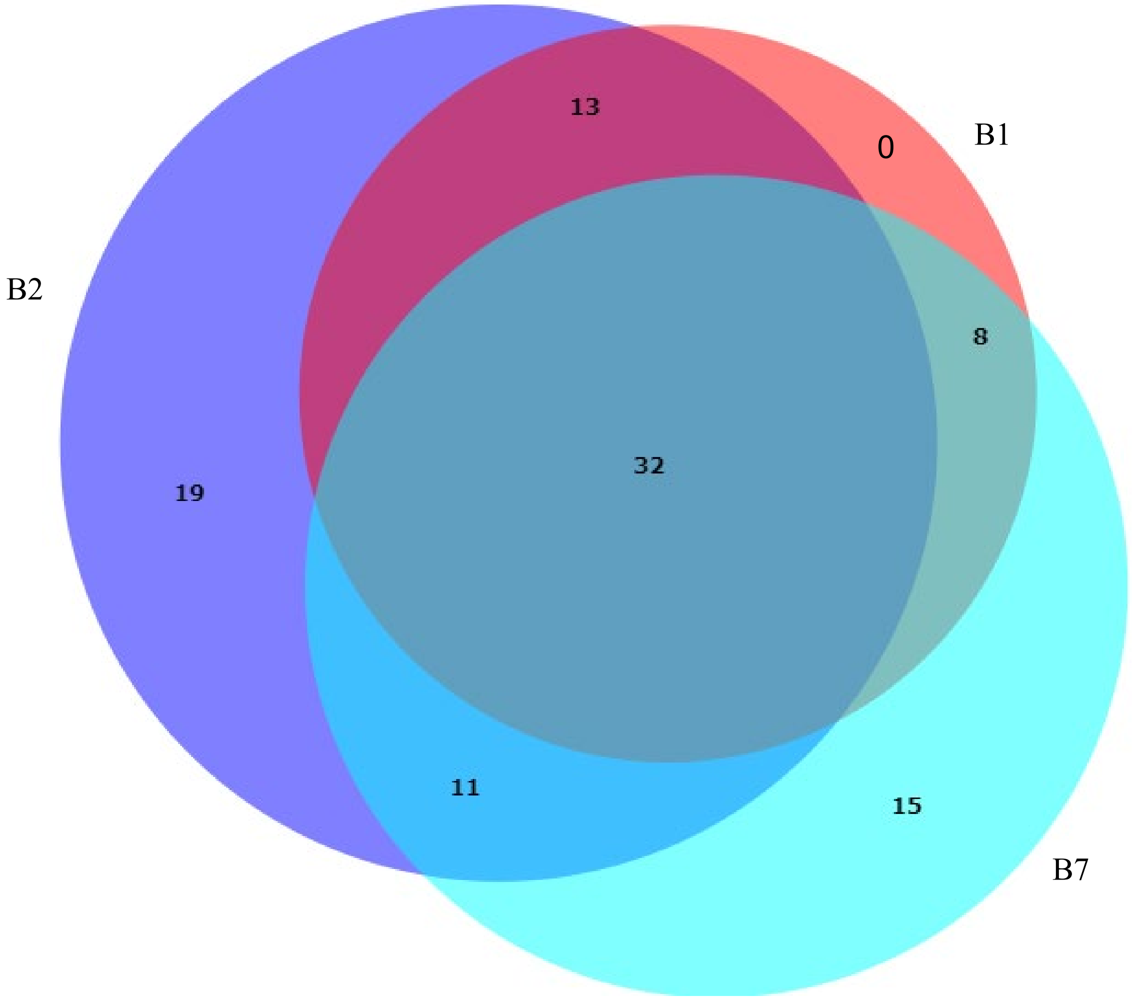

Fig. S10. Copy number analysis. (A) Odd ratios of copyKAT predicted aneuploid cells' enrichment in B cell subpopulations. We use fisher's exact test to compare the 1058 copyKAT predicted aneuploid cells with 518 B1 cells, 525 B2 cells, and 367 B7 cells respectively. The dots represent odd ratios, and the lines represent 95% confidence interval. (B) The Venn diagram of overlapping of significant differential copy number locus between three B cell subpopulation in CLL.

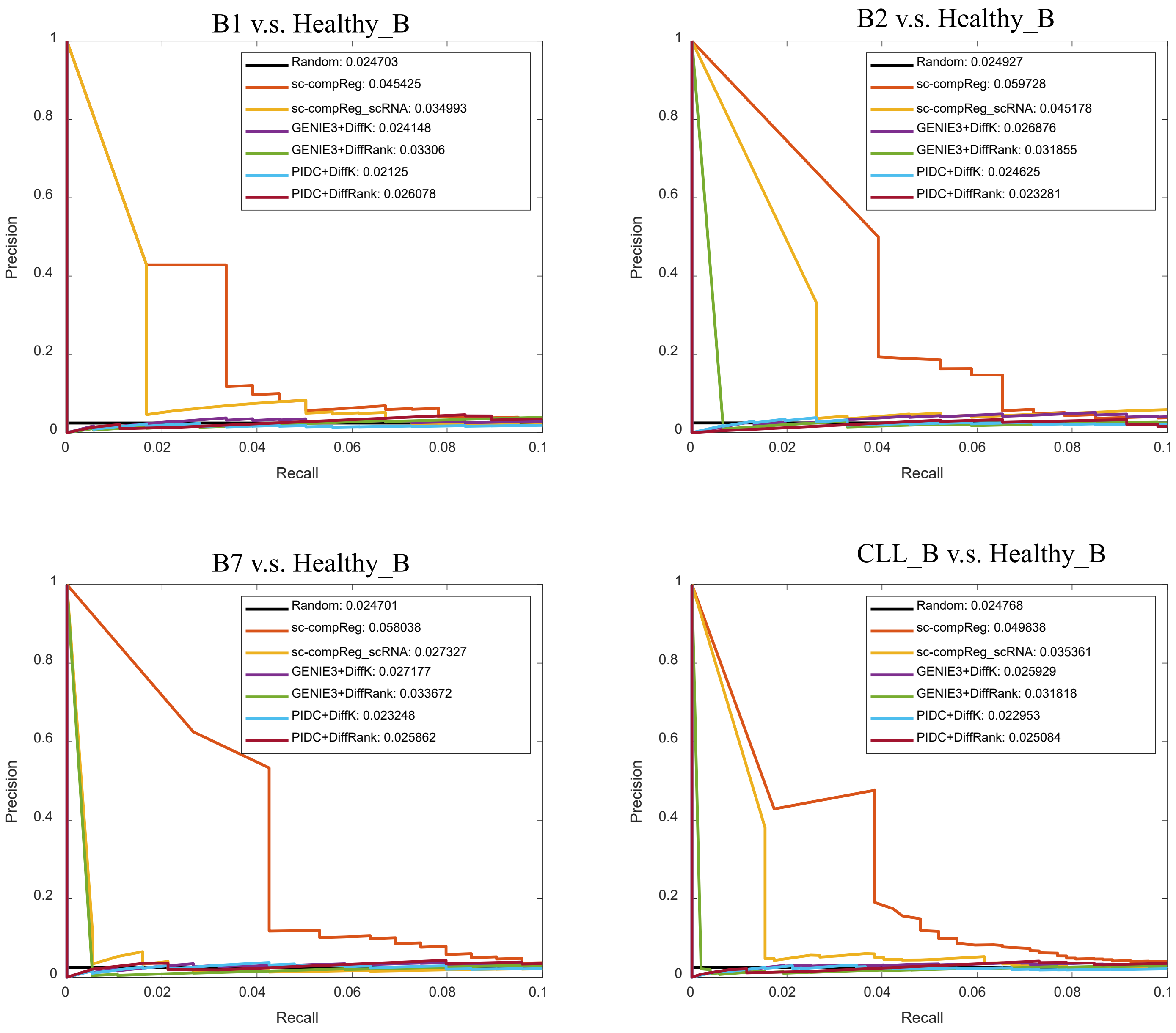

Fig. S11. Validation of differential regulatory network between three B cell subpopulation in CLL and healthy B cell. The ground truth is from the knowledge-based transcriptional regulatory network database Regnetwork. The figure shows the precision-recall curves. The lower right figure is after merging the prediction of 3 comparisons. As the collected ground truth is incomplete and also it contains regulation from non-relevant cellular context, the AUC is low. But it is still sufficient to compare the performance of different methods.

A

Rank	B1	B2	B7
1	TADA2B	ZADH2	TOX2
2	ZADH2	PLXNA3	ATF3
3	DAPK2	UBASH3B	FEZ1
4	CTRL	FZD3	RBPMS2
5	GPR34	TADA2B	COX4I2
6	ABCA9	ABCA9	BTNL9
7	GEN1	GIGYF1	NETO1
8	USP54	VPS13C	HES4
9	F8	TTN	PTMS
10	NETO1	AFF4	CCDC74A
11	TNS1	AHCTF1	GINS3
12	ZNF415	XYLT1	PHF19
13	XYLT1	RNF168	GTSF1L
14	FRMPD1	SMCHD1	RHOBTB3
15	ZBTB38	KIF21B	ATP6V0C
16	TWSG1	CYLD	RTN4R
17	PEX11G	NCOA3	ETV3
18	NAB2	FRMPD1	LRRC73
19	DOT1L	CAMK2D	GABRA4
20	RBMXL1	ZNF318	MS4A8

B

Category	GO term	Fold Change	P-value
Common	lipopolysaccharide-mediated signaling pathway	5.42	2.91E-04
Common	regulation of vascular smooth muscle cell proliferation	4.52	1.93E-04
Common	regulation of toll-like receptor signaling pathway	4.13	9.78E-05
B1	protein modification by small protein conjugation or removal	1.7	2.57E-07
B2	myeloid cell development	3.23	1.64E-04
B2	B cell differentiation	2.8	2.20E-05
B7	cell cycle process	1.52	1.67E-05

Fig. S12. Analysis of CLL B cell-specific genes. (A) List of top 20 B1, B2, and B7 specific genes. (B) Enriched GO terms in common specific genes, B1 specific genes, B2 specific genes, and B7 specific genes. We use Fisher's exact test to calculate p-values and the p-value showing here is before multiple testing adjustment.

A

```
50.6% identity in 472 residues overlap; Score: 934.0; Gap frequency: 7.0%

TOX2    1 MSENNQEFLLANQTYSGES--NEDYEIPPITPPNLPDTSLLHLVDHETGYHSLCHSLPHN
TOX    44 MTEPSQDYVPASQSYPGPSLESEDFNIPPITPPSLPDHSLVHLNEVESGYHSLCHPMNHN
          * *  *    * * * * *   **  ******* *** ** **   * *******   **

TOX2   59 GLLPPYSYQNMDLPALMVSNMLNQEGHLLSGHLPTIQELVHSEGFQSDSNHQVPLI----
TOX   104 GLLP-FHPQNMDLPEITVSNMLGQDGTLLSNSISVMPDIRNPEGTQYSSHPQMAAMRPRG
          ****    ******   ***** * * ***            ** *  *  *

TOX2  115 ------SRPGMLPN-HMSTLSQSQLISQIGVRSG---VTHGSPSPPGSKSATPSPSSSTQ
TOX   163 QPADIRQQPGMMPHGQLTTINQSQLSAQLGLNMGGSNVPHNSPSPPGSKSATPSPSSSVH
                  *** *     *  ****  * *   *   * * *****************

TOX2  165 EEETDSFSKISA-EKRPSTDMGKKPKTQKKKKKKDPNEPQKPVSAYALFFRDTQAAIKGQ
TOX   223 EDEGDDTSKINGGEKRPASDMGKKPKTPKKKKKKDPNEPQKPVSAYALFFRDTQAAIKGQ
          * * *  ***   ****  ******** ********************************

TOX2  224 NPNATFGDVSKIVASMWDSLGEEQKQAYKKRTEAAKKEYLKALAAYRASLVSKSYSEQVE
TOX   283 NPNATFGEVSKIVASMWDGLGEEQKQVYKKKTEAAKKEYLKQLAAYRASLVSKSYSEPVD
          ******* ********** ******* *** ********** *************** *

TOX2  284 PKTAHSSHPSHMFPAK-QPLYNVQPPTSSPYPYLPSSEMQSYHGSPHTGLTRTLTSK---
TOX   343 VKTSQPPQLINSKPSVFHGPSQAHSALYLSSHYHQQPGMNPHLTAMHPSLPRNIAPKPNN
           **          *                   *     *       *  * *    *

TOX2  340 QM-----LSNLSASPPPSFQISPPLHQQLALCHPPNSLMNQPMNLQHVPQQSVMSHQMSL
TOX   403 QMPVTVSIANMAVSPPPPLQISPPLHQHLNMQQHQPLTMQQPLGNQLPMQVQSALHSPTM
          **       *   ****  ******** *         * **   *   *     *

TOX2  395 QVQPPMSSPPGQQNFSHIQSEFQSSSQS-----PGPSNPPISTSDWDEDYCN
TOX   463 Q-QGFTLQPDYQTIINPTSTAAQVVTQAMEYVRSGCRNPPPQPVDWNNDYCS
          * *     *  *          *   *       *  ***    **  ***
```

B

	TOX-target	Non TOX-target	Total
Co-expressed with TOX2	**26**	440	466
Not Co-expressed with TOX2	504	17577	18081
Total	530	18017	18547

Fisher exact test
P-value : 9.6069e-04
Odd Ratio : 2.0608
ConfidenceInterval: [1.3740 3.0909]

C

Enriched motif on TOX dependent open regions (GSE132986)

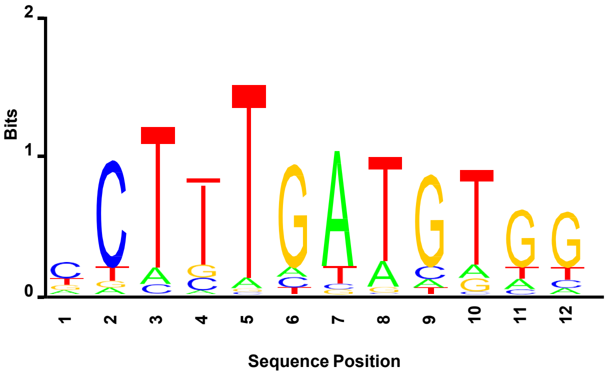

Tcf4(HMG)/Hct116-Tcf4-ChIP-Seq (SRA012054)/Homer

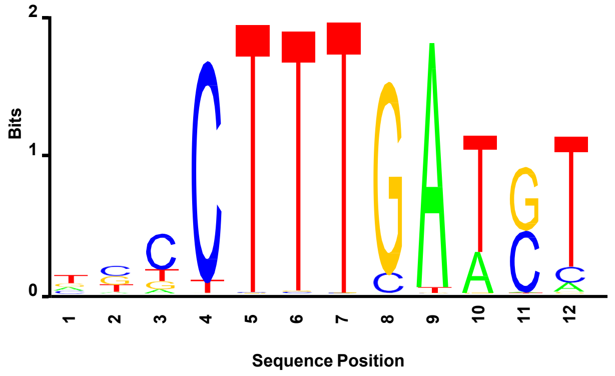

Fig. S13. TOX2 motif analysis. (A) Comparison of TOX and TOX2 protein sequence. The red box part represents the DNA binding domain. (B) Comparison of TOX ChIP-seq target with TOX2 co-expressed genes. We use Fisher's exact test to compare them. The testing results like p-values, odd ratios, and 95% confidence interval are shown. (C) Comparison of the de novo motif enriched in the TOX dependent region with a known motif of TCFL2.

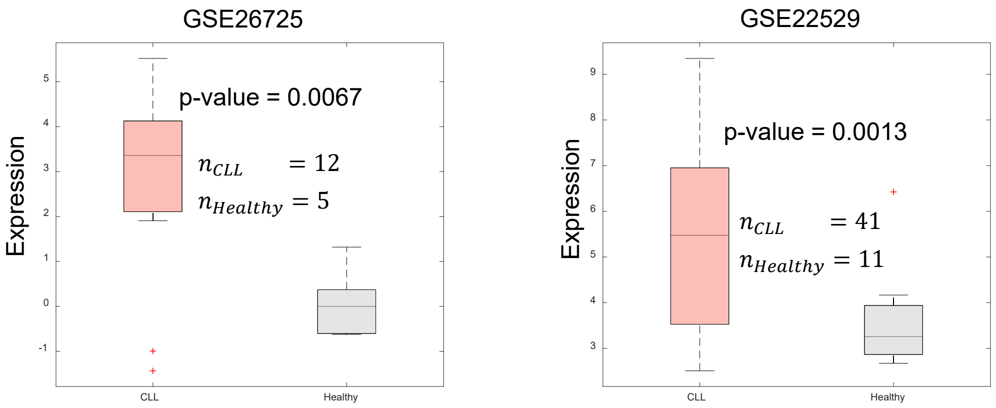

Fig. S14. Validation of TOX2 on two independent case-control studies. The central mark indicates the median, and the bottom and top edges of the box indicate lower and upper quartiles. P-values are calculated from a one-tailed two-sample t-test without doing multiple testing adjustment. The central mark indicates the median, the bottom and top edges of the box indicate lower and upper quartiles, the whiskers extend to the most extreme data points not considered outliers, and the outliers are plotted individually using the '+' symbol.

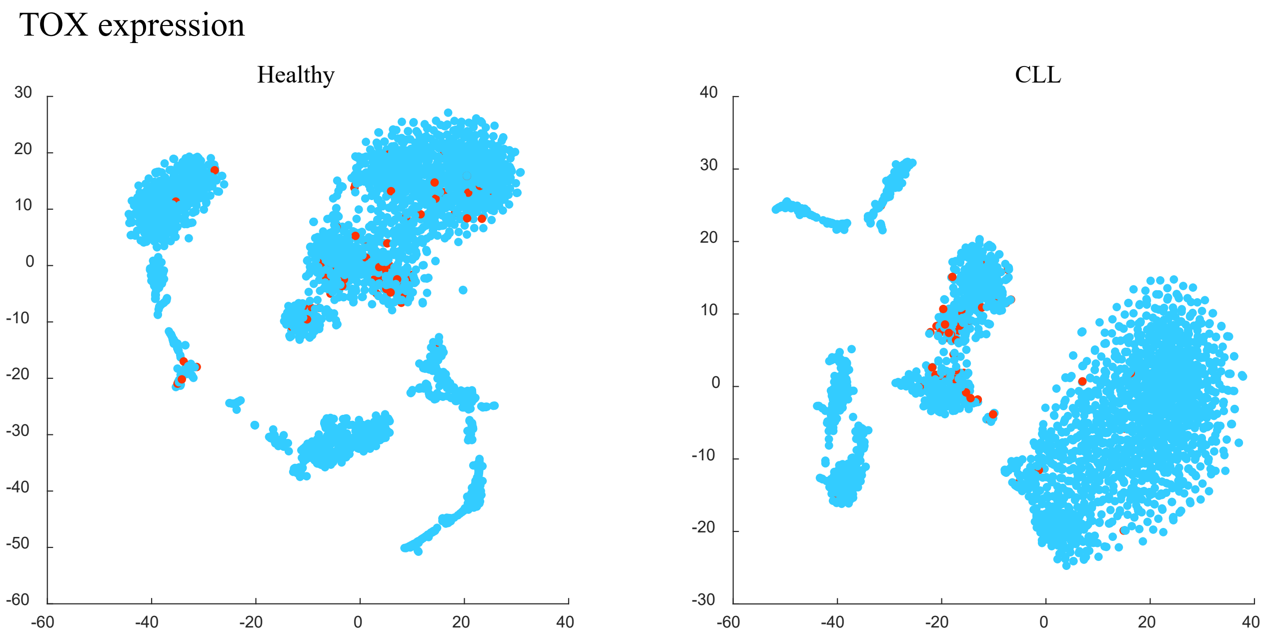

Fig. S15. The t-SNE plot shows the expression of TOX in the healthy donor (left) and the CLL donor (right). Red dots represent TOX expressed cells.

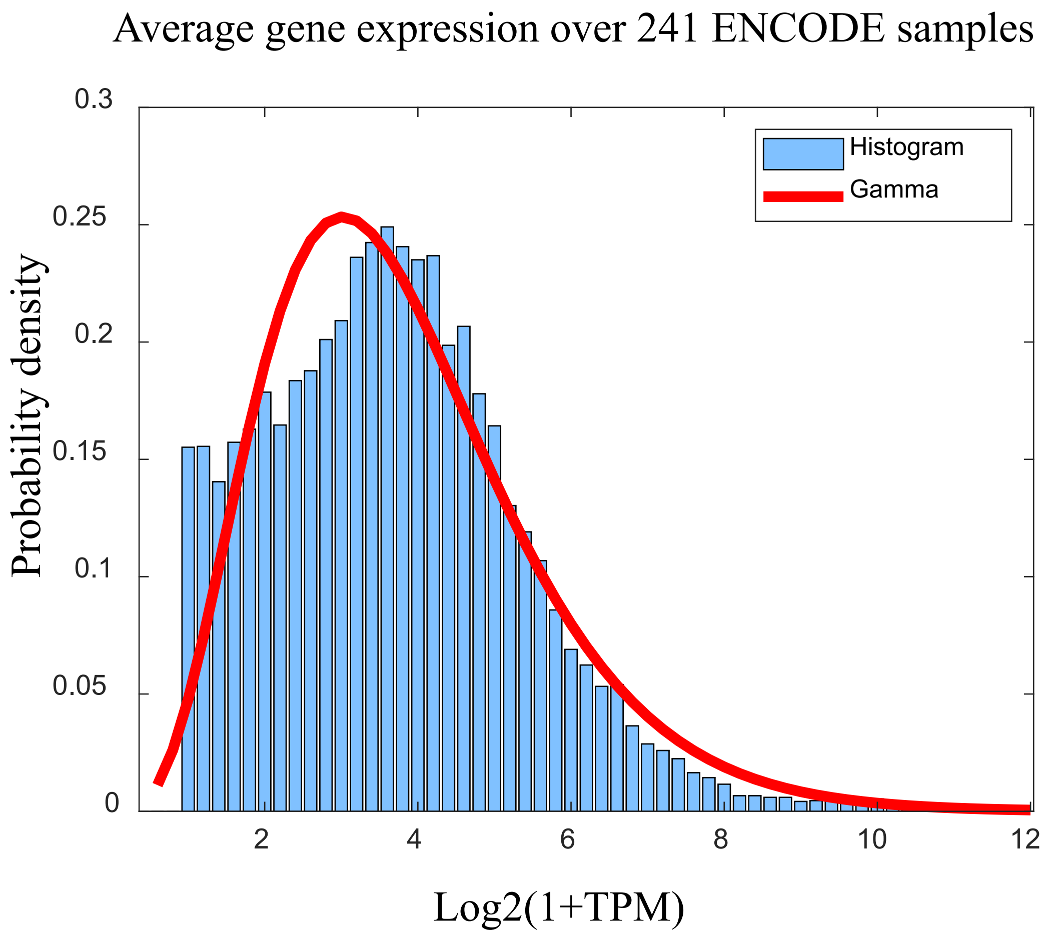

Fig. S16. Distribution of average gene expression follows a Gamma distribution.

References

1. Stuart T, *et al.* (2019) Comprehensive integration of single-cell data. *Cell* 177(7):1888-1902. e1821.
2. Scott AC, *et al.* (2019) TOX is a critical regulator of tumour-specific T cell differentiation. *Nature* 571(7764):270.
