# Supplementary material for: Sc-compReg enables the comparison of gene regulatory networks between conditions using single-cell data
Source: Nat Commun. 2021 Aug 6;12:4763. doi: 10.1038/s41467-021-25089-2 (PMC8346476; doi:10.1038/s41467-021-25089-2)
Supplement: Supplementary file 3 — Description of Additional Supplementary Files [file 41467_2021_25089_MOESM3_ESM.pdf]

Description of Additional Supplementary Files

File Name: Supplementary Data 1
Description: Abnormal copy number regions in three B cell subpopulations from CLL compared to healthy B cell.
