# Supplementary material for: Sc-compReg enables the comparison of gene regulatory networks between conditions using single-cell data
Source: Nat Commun. 2021 Aug 6;12:4763. doi: 10.1038/s41467-021-25089-2 (PMC8346476; doi:10.1038/s41467-021-25089-2)
Supplement: Supplementary file 5 — Reporting Summary [file 41467_2021_25089_MOESM5_ESM.pdf]

nature portfolio

Corresponding author(s): Wing Hung Wong

Last updated by author(s): Jun 17, 2021

Reporting Summary

Nature Portfolio wishes to improve the reproducibility of the work that we publish. This form provides structure for consistency and transparency in reporting. For further information on Nature Portfolio policies, see our Editorial Policies and the Editorial Policy Checklist.

Statistics

For all statistical analyses, confirm that the following items are present in the figure legend, table legend, main text, or Methods section.

n/a	Confirmed	
☐	☒	The exact sample size (*n*) for each experimental group/condition, given as a discrete number and unit of measurement
☐	☒	A statement on whether measurements were taken from distinct samples or whether the same sample was measured repeatedly
☐	☒	The statistical test(s) used AND whether they are one- or two-sided<br>*Only common tests should be described solely by name; describe more complex techniques in the Methods section.*
☐	☒	A description of all covariates tested
☐	☒	A description of any assumptions or corrections, such as tests of normality and adjustment for multiple comparisons
☐	☒	A full description of the statistical parameters including central tendency (e.g. means) or other basic estimates (e.g. regression coefficient) AND variation (e.g. standard deviation) or associated estimates of uncertainty (e.g. confidence intervals)
☐	☒	For null hypothesis testing, the test statistic (e.g. *F*, *t*, *r*) with confidence intervals, effect sizes, degrees of freedom and *P* value noted<br>*Give P values as exact values whenever suitable.*
☒	☐	For Bayesian analysis, information on the choice of priors and Markov chain Monte Carlo settings
☒	☐	For hierarchical and complex designs, identification of the appropriate level for tests and full reporting of outcomes
☐	☒	Estimates of effect sizes (e.g. Cohen's *d*, Pearson's *r*), indicating how they were calculated

Our web collection on statistics for biologists contains articles on many of the points above.

Software and code

Policy information about availability of computer code

Data collection	no software was used
Data analysis	ATAC FASTQs were processed using the `cellranger-atac count` pipeline (pre-release 1.0 version) with the option `--force-cells 4200`. We develop software sc-comReg to analyze the data, which is available on github: https://github.com/SUwonglab/sc-compReg. The CoupledNMF method is also implemented in the sc-CompReg software which is based on NMF and Hungarian algorithm. We also use Cell Ranger and Seurat V3 to cluster the single cell data and use PECA2 to infer the gene regulatory network.

For manuscripts utilizing custom algorithms or software that are central to the research but not yet described in published literature, software must be made available to editors and reviewers. We strongly encourage code deposition in a community repository (e.g. GitHub). See the Nature Portfolio guidelines for submitting code & software for further information.

Data

Policy information about availability of data

All manuscripts must include a data availability statement. This statement should provide the following information, where applicable:

- Accession codes, unique identifiers, or web links for publicly available datasets
- A description of any restrictions on data availability
- For clinical datasets or third party data, please ensure that the statement adheres to our policy

All processed sequencing data generated in this study have been submitted to the NCBI Gene Expression Omnibus (GEO; https://www.ncbi.nlm.nih.gov/geo/) under accession number GSE159417.

March 2021

Field-specific reporting

Please select the one below that is the best fit for your research. If you are not sure, read the appropriate sections before making your selection.

- [x] Life sciences
- [ ] Behavioural & social sciences
- [ ] Ecological, evolutionary & environmental sciences

For a reference copy of the document with all sections, see nature.com/documents/nr-reporting-summary-flat.pdf

Life sciences study design

All studies must disclose on these points even when the disclosure is negative.

Sample size	4200 cells with scATAC-seq and 2760 cells with scRNA-seq from healthy donor; 4203 cells with scATAC-seq and 2661 cells with scRNA-seq from CLL donor. The sample size (the number of cells) is the standard output of the cell ranger software.
Data exclusions	272 and 423 cells from healthy and CLL donors scATAC-seq data are removed. We develop a new method for scATAC-seq data to identify the bad cells by calculating the percentage of reads in peaks. The method are described in the supplementary note.
Replication	We didn't attempt to replicate the experiment.
Randomization	Randomization is not relevant to this study as we only have one healthy donor and one disease donor.
Blinding	Investigators are not blinded to group allocation during the data collection and analysis. We don't think this will affect the results too much as we are using standard protocol and software in default parameters.

Reporting for specific materials, systems and methods

We require information from authors about some types of materials, experimental systems and methods used in many studies. Here, indicate whether each material, system or method listed is relevant to your study. If you are not sure if a list item applies to your research, read the appropriate section before selecting a response.

Materials & experimental systems

n/a	Involved in the study
[x]	[ ] Antibodies
[x]	[ ] Eukaryotic cell lines
[x]	[ ] Palaeontology and archaeology
[x]	[ ] Animals and other organisms
[ ]	[x] Human research participants
[x]	[ ] Clinical data
[x]	[ ] Dual use research of concern

Methods

n/a	Involved in the study
[x]	[ ] ChIP-seq
[x]	[ ] Flow cytometry
[x]	[ ] MRI-based neuroimaging

Human research participants

Policy information about studies involving human research participants

Population characteristics	Bone marrow mononuclear cells (BMMCs) from one healthy donor and one patient with chronic lymphocytic leukemia (CLL) are included.One donor was a 93 year old non-Hispanic white male with newly-diagnosed chronic lymphocytic leukemia, and the other individual was healthy but otherwise unknown.
Recruitment	Samples were purchased from AllCells. Donor pool information is available at https://www.allcells.com/cell-tissue-procurement/the-donor-difference/
Ethics oversight	Bone Marrow and/or Peripheral Blood Collection From Disease-Specific Donors for the Research Market (7000-SOP-078), and Bone Marrow Collection from Healthy Donors for the Research Market (7000-SOP-046) were approved by Alpha IRB

Note that full information on the approval of the study protocol must also be provided in the manuscript.
